# Malaria in Africa Can Be Eliminated

**DOI:** 10.4269/ajtmh.2011.11-0529

**Published:** 2011-10-01

**Authors:** Carlos C. Campbell, Richard W. Steketee

**Affiliations:** Malaria Control Program, PATH, Seattle, Washington

## Abstract

A concerted effort to control malaria in Africa has produced dramatic reductions in childhood death in the past decade. This early success has prompted the global community to commit to eradication of malaria deaths and eventually all transmission. Evidence suggests that this is a feasible goal using currently available interventions, augmented with newer tools such as vaccines, which are in development. Malaria deaths are entirely preventable now, and our sustained political and financial commitment to continue to prevent these deaths hangs in the balance.

We are witnessing major successes in fighting malaria—a disease that, until recently, killed over 1 million people each year, mainly African children. The accomplishments have been impressive. The scale up for impact (SUFI) strategy—rapidly delivering malaria prevention interventions to achieve coverage of most or all at-risk populations—has been endorsed by the global Roll Back Malaria (RBM) Partnership,^1^ and has become the national malaria control standard in the Africa region. An increasing number of African countries, led by their national malaria partnerships, are distributing millions of long-lasting insecticide-treated mosquito nets (LLINs), spraying household walls with insecticides, and rolling out new diagnostics and effective medicines nationwide to strengthen the management of malaria infections. The package of interventions, especially the LLINs, has proven to be a powerful arsenal capable of saving many lives. Many countries have recently documented a fall in childhood deaths in the range of 20–25%, and it is estimated that more than 1 million African children are alive today that otherwise would have died of malaria^2^; however, it is too early to celebrate.

This dramatic success has created what will become the generational challenge for malaria control. Scale-up campaigns have jump-started malaria control, bringing down malaria transmission to levels where deaths are largely prevented and malaria is no longer a pervasive drain on health services. Although as malaria declines, so may commitment to fight the disease. With the malaria problem seemingly “solved,” national governments and funding agencies can easily turn their focus—and wallets—to other pressing health priorities.

As SUFI was becoming the rallying cry for national malaria programs, eradication was placed back on the global agenda as a long-term vision in 2007 (Gates Foundation Malaria Forum, October 2007, Seattle WA). The memory of the earlier Malaria Eradication Program of the 1950s and 60s^3^ was invoked—both its successes (malaria elimination was certified in many countries) and its failures (elimination faltered because of heavy reliance on indoor insecticide spraying and evolving insecticide resistance resulting in malaria resurgence in many areas). The call for eradication sparked some skepticism initially, but has overall generated enthusiasm and commitment. The RBM Global Malaria Action Plan (GMAP) charted the pathway from scale up to elimination. The World Health Organization (WHO)^4^ and the Malaria Elimination Group^5^ described approaches to elimination. And, with the suggestion that new and better tools would be required, the Malaria Eradication Research Agenda (malERA) Group^6^ detailed the research agenda to generate new tools and strategies.

The complex challenges ahead to fully eliminate malaria from Africa are becoming clearer. Although the GMAP embraced both SUFI and elimination, the middle ground— termed “sustained control”—highlighted the need for continued work to preserve the progress in achieving high coverage but did not address how a national program could actually transition to elimination. Unfortunately, this means that the malaria control community and the many countries that have experienced marked progress do not have clarity or consensus, on the next steps.

We suggest that there are discrete steps on the path between scale up and elimination and that, in most places, these steps can be taken using existing control methods (LLINs, indoor residual spraying, diagnostics and effective antimalarial medicines). Adaptation of these tools, guided by a focused strategy that continuously evolves to address the dynamic challenges of reducing malaria transmission, can ultimately result in countries achieving zero transmission. Zero malaria transmission would mean a true end to the plague of malaria illness and death.

The first step is to finish the scale-up work. Despite huge efforts, the current malaria control strategies have not yet been deployed to fully cover the populations at risk. And, because the child who was protected under an LLIN last night must sleep under one each successive night, the culture of malaria prevention must become part of the fabric of life in every community.

The second step is to gain efficiencies in delivering the preventive and case management components of the strategy. This step includes strengthened management and supply chain systems that make it easier to anticipate and fill program gaps in a timely manner or before they actually become gaps—focusing on human resource needs, commodity supplies, and local data to guide program implementation. This work both ensures full scale up and optimizes the use of available resources.

The third step is to further reduce malaria transmission. The program gains that have been achieved with the implementation of SUFI have resulted in a major (~10-fold) reduction in malaria transmission intensity^7^; in many areas, remaining levels of transmission are still too high and must be brought down another 10-fold or more to reach levels that fulfill pre-elimination or elimination criteria. Because the first transmission reductions were largely accomplished through killing mosquitoes, the remaining parasites mostly reside in people. Clearing these infections requires strategies to systematically find and kill parasites in the human population. This is not simply an improved management of symptomatic infections because many infected (and transmitting) people are asymptomatic; to further reduce transmission, we must find and cure all infected people.

Clearing all malaria infections is only possible with access to real-time data on where the residual infections are today and in the future. All countries striving for elimination will need to know when elimination has occurred and will need surveillance, diagnostic capability, and monitoring and evaluation systems that have sufficient reach and quality to provide that information in real time.

There is accumulating program experience in a diverse range of African countries suggesting that maintaining high LLIN usage combined with aggressive malaria infection detection and drug treatment to completely kill off all detected malaria infections can break the chain of malaria transmission in communities.^8^ This approach is currently being tested in national programs in a number of African nations; soon these efforts will generate solid evidence on the extent to which it is effective in eliminating malaria deaths and reducing transmission.

Experience from decades past has taught that the path to elimination has identifiable steps, but the progression is not entirely predictable across all countries. Attempts at eradicating malaria in the mid-1960s were rapidly successful in many countries, but progress was much slower and more complex in other areas, and the effort was eventually abandoned. Donor fatigue led to a loss of funding and commitment to control malaria. The recipe for success this time must include community involvement and ownership, local and national willingness (including growing domestic funding) to persevere until the task is complete, and unwavering global support. We must embrace this as a learning process that will require adaptive science to achieve elimination.

It has been suggested that malaria elimination is too ambitious a goal, impossible to achieve with current interventions and available financing, and that we must wait for some future tool—such as a vaccine that blocks malaria transmission—to aspire to stopping transmission. Alternative goals have been suggested; for example, to attempt to maintain malaria transmission at low levels whereby malaria deaths are few but the program costs are recurrent with no definable end point. Some have even suggested that Africa should just live with some level of malaria, as if that is not what Africa has been forced to do for time in memoriam.

Malaria control remains one of the best investments in global health: malaria deaths can be eliminated now with currently available interventions. We have vastly more powerful tools than ever before, we have comprehensive methods of collecting data to inform our work, and national governments are leading the charge, leveraging external assistance to dramatically reduce malaria illnesses and deaths. New and potentially more powerful interventions such as malaria vaccines will be available in the foreseeable future. However, we must refuse to let history repeat itself.

We must reach beyond the relatively easy successes of SUFI to address the next steps for malaria control in Africa; our shared ambition must be elimination not only of deaths but the permanent end of transmission. This will require sustaining and even increasing the unprecedented financial resources invested in the last decade,^9^ despite competing health priorities, and committing to the challenge until the job is done. Mothers and their children throughout Africa deserve our assurance that we will not settle for less.
